# Survey of nutritional intake status in college baseball players

**DOI:** 10.1080/15502783.2025.2459090

**Published:** 2025-02-03

**Authors:** Yoko Iio, Hana Kozai, Mamoru Tanaka, Yukihiro Mori, Manato Seguchi, Yuka Aoyama, Morihiro Ito

**Affiliations:** a Chubu University, Graduate School of Life and Health Sciences, Kasugai, Japan; b Chubu University, Department of Lifelong Sports and Health Sciences, College of Life and Health Sciences, Kasugai, Japan; c Chubu University, Department of Food and Nutritional Sciences, College of Bioscience and Biotechnology, Kasugai, Japan; d Chubu University, Department of Nursing, College of Life and Health Sciences, Kasugai, Japan; e Chubu University, Department of Clinical Engineering, College of Life and Health Sciences, Kasugai, Japan; f Chubu University, Department of Biomedical Sciences, College of Life and Health Science, Kasugai, Japan

**Keywords:** Nutrition intake, college baseball players, Food Frequency Questionnaire (FFQ)

## Abstract

**Background:**

Diet is closely related to exercise performance. To improve athletes’ performance and manage their condition, it is important to get sufficient energy and various nutrients. Thus, it is necessary that athletes understand their nutritional intake status to improve performance and maintain health. This study aimed to explore the nutritional intake status of college baseball players using the Food Frequency Questionnaire (FFQ). Furthermore, the characteristics of their nutritional intake status with respect to athletic performance were evaluated. The result of this studyprovide an opportunity for many under-developed college athletes with irregular lifestyles to recognize and improve their nutritional problems.

**Methods:**

In October 2022, a questionnaire survey of 116 male members of a college baseball club was conducted. Of whom, 100 (94.3%) members responded to the survey and 92 (92.0%) provided valid responses. The survey items included basic characteristics such as college grade and type of living arrangement, and information on living conditions, e.g. whether the participant ate breakfast. Nutritional intake was evaluated using the FFQ. Players were divided into the first (regular players in official games), second (bench players in official games), third (players who may join the second or higher team in the future), and fourth teams (players who do not belong to the first to third teams); these categories were used as a marker of performance level. The Kruskal-Wallis test was used to analyze the association between the performance levels of baseball players and the intake of each nutrient and food group obtained by the FFQ. For items that showed a significant association, inter-group comparison was performed using the Dunn-Bonferroni method.

**Results:**

Carbohydrate intake was greater in the second team compared with the third and fourth teams; saturated and monounsaturated fatty acid intake was higher in the third team compared with the fourth team. Calcium, zinc, copper, manganese, insoluble dietary fiber, iodine, and molybdenum intake was higher in the second team compared with the fourth team. Intake of grains, sugar, dairy, and total energy was significantly higher in the second team compared with the fourth team. However, the protein intake ratio was significantly lower in the second team compared with the fourth team. Overall, energy deficiency and associated deficiencies in protein, fat, and carbohydrate were observed, in addition to dietary fiber and calcium deficiencies. The intake of several food groups appeared inadequate, such as potatoes, beans, vegetables, fruits, eggs, milk, and fats.

**Conclusions:**

The study showed deficiencies in the amount of energy and nutrients such as protein, fat, and carbohydrate in college baseball players. Differences in the intake of carbohydrate, calcium, and insoluble dietary fiber among different performance levels were observed, with significantly higher intake of carbohydrate, calcium, and insoluble dietary fiber in the second team. Implementing organized and strategic remedial measures and athletes’ identification of nutritional problems are vital to overcome nutritional and energy deficiencies. This study provides useful information for the development of strategies to support nutritional intake in college baseball players.

## 1. Introduction

The relationship between sports performance and nutrition has been established and discussed in detail [1]. An adequate nutrient supply is essential to provide fuel to support physical activity, adaptation to training, and recovery. Similarly, adequate hydration is important to prevent dehydration, which can reduce exercise capacity [2,3]. Statements on nutrition and exercise capacity issued by the American College of Sports Medicine [2], International Olympic Committee [4], and International Society of Sports Nutrition [1] indicate that athletes need more energy and nutrients than the general population and should pay special attention to the timing of nutrient intake [1,2]. Thus, research has established the importance of adequate intake of energy and various nutrients to improve athletes’ performance and manage their condition [2], with further research on this topic being ongoing.

Protein requirements increase for the repair and synthesis of muscle proteins that are broken down during exercise; therefore, the recommended amount of protein for athletes is 1.2–2.0 g/kg body weight/day [1,2]. Carbohydrates play an important role in providing energy, maintaining performance, and ensuring recovery for athletes [1]. The need for carbohydrates before and after intense training and competitions and based on the amount of exercise is well known. Fats are also an important source of energy when muscle glycogen is depleted, which is important for improving endurance and performance in high-intensity sports [5]. Moreover, micronutrients are also important. Intake of the vitamin B complex, whose members perform important functions in energy metabolism, is considered essential for players of sports such as soccer, where the energy expenditure is enormous [6]. Baseball is less aerobic than many sports, but there are multiple games during the season that last more than 3 hours, which requires endurance and quick recovery from fatigue [7]. Therefore, adequate intake of vitamin B complex via well-balanced meals is important. Vitamin C and vitamin E have a role in reducing oxidative stress, which increases with exercise, and are thus important factors for athletes [8,9]. Iron helps transport oxygen and affects endurance exercise performance, and is also essential for including oxidative stress and maintaining immune function [8,9]. In addition, calcium and magnesium are essential for bone formation and maintenance and for maintaining and improving performance as they are involved in normal muscle contraction [9].

Furthermore, studies have mentioned the possibility of vitamin D deficiency in athletes [10], and one study has reported that the intake of antioxidants present in vegetables and fruits is effective in reducing oxidative stress caused by exercise [11]. Thus, the importance of nutritional management in maintaining condition and physical preparation cannot be underestimated, e.g. replenishing carbohydrate expended during daily training and games, and protein for skeletal muscle synthesis [12]. However, studies have shown that athletes often fail to meet these dietary recommendations [13]. Therefore, intake of a balanced diet containing abundant nutrients without missing meals is essential. Athletes are required to self-manage their life activities, which include eating well-balanced meals, as well as their training. Compared with high-performance professional athletes, college athletes receive less support for competition and life; they must balance and manage their studies and competitions on their own, which is a daunting task.

Although studies have evaluated the nutritional and lifestyle habits of high-performance athletes, only a few have examined college students playing a single sport. Several recent studies on nutrition have used the Food Frequency Questionnaire (FFQ) [14], which is reportedly superior to other dietary survey methods [15]. This study aimed to investigate the nutritional intake status of college baseball players using the FFQ. Furthermore, we evaluated the characteristics of their nutritional intake status in terms of athletic performance. Therefore, the results of the study provide the rationale for the utility of evaluating food habits, with a focus on nutritional intake, to improve athletic performance. Moreover, these results also provide an opportunity for many college-level athletes, whose lifestyles tendto be irregular and who receive little support, to recognize their nutritional problems and improve their nutritional status.

## 2. Materials and methods

### 2.1. Study design and data collection

For 1 month from late October 2022, this cross-sectional study conducted a questionnaire survey of 116 male students belonging to the baseball club of University A to determine the nutritional intake status and characteristics of players. A total of 100 (94.3%) players responded to the survey, and 92 (92.0%) players provided valid responses. The autumn collegiate baseball league season, in which the surveyed team participated, runs from September to mid-October. The survey was conducted during the postseason, shortly after the end of the autumn league season.

Data collection was performed using an anonymous web-based questionnaire designed using Google Forms (Google LLC, Mountain View, CA, USA). Printed material providing information about the study was distributed to recruit active baseball players. Students who agreed to participate used a mobile phone to scan a QR code for the Google Forms URL to access and answer the web questionnaire survey.

Statistical power analysis was performed using G*Power 3.1.9.7 (Heinrich Heine University, Dusseldorf, Germany) to validate the sample size of the study. With an effect size of 0.4, a significance level of 0.05, a sample size of 92, and four study groups, the power of the test was 0.80. Similarly, with a sample size of 90 and three study groups, the power of the test was 0.93.

The present study was approved by the Ethics Review Committee of Chubu University (Kasugai, Japan; Approval Number: 20220055).

### 2.2. Survey items

#### 2.2.1. Basic items

The survey comprised 201 items. Basic characteristics such as the college grade and type of living arrangement, and information on living conditions, e.g. whether the participant ate breakfast, were collected (Table 1). There were three choices for living arrangement, viz, living in the parents’ home, living alone, or living in a dormitory. Two meals, viz. breakfast and dinner, are provided in the dormitory on weekdays. In the baseball club, coaches evaluate the performance of the players and assigned them to the first to fourth teams in descending order of their performance level. The first team consists of regular players in official games, the second team consists of bench players in official games, the third team consists of players who may join the second or higher team in the future, and the fourth team consists of other players. Participants were also asked which category they belonged to, and we used the category as a marker of performance level.

**Table 1.** Participants’ characteristics.

	N	%
Grade		
Freshman	34	37.0%
Sophomore	32	34.8%
Junior	26	28.3%
Residence style		
Parents’ home	48	52.2%
Boarding house	38	41.3%
Dormitory	6	6.5%
Performance level		
First team	12	13.0%
Second team	14	15.2%
Third team	34	37.0%
Fourth team	32	34.8%
Frequency of eating breakfast		
Twice a week or less	4	4.3%
3–4 days a week	8	8.7%
Approximately 5 days a week	10	10.9%
Daily	70	76.1%
BMI (kg/m^2^)		
18.5 − 25.0 (normal weight)	53	57.6%
25.0 − 30.0 (first-degree obesity)	37	40.2%
30.0 − 35.0 (second-degree obesity)	2	2.2%

#### 2.2.2. Nutritional intake status

The FFQ was used to assess the participants’ nutritional intake status [16]. The FFQ was developed by the Japan Public Health Center-based Prospective Study for the Next Generation (JPHC-NEXT) to determine the usual eating and nutritional intake status in a single survey and has been used in several previous studies [17–20]. Detailed and simplified versions are available, and the present study used the former, which can determine the intake of each nutrient, food group, and type of beverage.

### 2.3. Statistical analysis

For all questionnaire items, the grand total was calculated. In the present study, we hypothesized that the baseball players’ performance levels and attributes/living condition would be associated with their nutritional intake status. To test this hypothesis, the Kruskal-Wallis test was used to analyze the associations of baseball players’ performance levels with the intake of each nutrient and food group obtained using the FFQ. For items that showed a significant association, intergroup comparison was performed using the Dunn-Bonferroni method (Tables 2–4). The dependent variables were performance levels and attributes/living conditions, and the explanatory variables were the intake of each nutrient and each food group. The significance level was set at *p* < 0.05 for all items. All statistical analyses were performed using SPSS version 27 IBM software (Statistical Package for Social Science, Chicago, Illinois, USA).

**Table 2.** Association between baseball players characteristics and total calories or energy-providing nutrient balance.

			Energy (kcal)			Protein ration (%E)			Fat ration (%E)			Saturated fatty acid ration (%E)			Carbohydrate ration (%E)		
	n	%	Median	IQR	*p*	Median	IQR	*p*	Median	IQR	*p*	Median	IQR	*p*	Median	IQR	*p*
Total	92	100	2077.4	(1959.0–2262.4)	*N/A*	13.3	(12.9–13.6)	*N/A*	24,5	(23.8–25.8)	*N/A*	7.7	(7.4–8.0)	*N/A*	62.0	(60.7–63.0)	*N/A*
Residence Style																	
Parents’ home	48	52.2%	2154.6	(1975.4–2300.0)	0.227	13.3	(12.9–13.6)	0.340	25.0	(23.7–25.8)	0.829	7.8	(7.4–8.0)	0.896	61.8	(60.8–63.2)	0.776
Boarding house	38	41.3%	1942.7	(1942.7–2222.3)		13.3	(12.7–13.6)		24.5	(23.6–25.6)		7.6	(7.4–8.0)		62.1	(60.7–62.9)	
Dormitory	6	6.5%	1960.4	(1960.4–2029.7)		13.6	(13.2–14.1)		24.4	(24.1–25.9)		7.6	(7.5–8.2)		62.2	(60.0–62.5)	
Performance level																	
First team	12	13.0%	2055.1	(1868.4–2233.5)	0.013^a^	13.5	(12.9–13.7)	0.015^b^	24.2	(23.2–25.6)	0.160	7.6	(7.3–8.0)	0.213	62.2	(60.7–63.9)	0.102
Second team	14	15.2%	2304.2	(2111.2–2471.8)		12.9	(12.3–13.2)		23.9	(23.6–25.4)		7.5	(7.3–7.8)		62.9	(61.4–63.7)	
Third team	34	37.0%	2085.8	(1967.4–2237.1)		13.3	(13.0–13.5)		25.0	(23.9–26.3)		7.7	(7.5–8.2)		61.8	(60.3–62.8)	
Fourth team	32	34.8%	2009.3	(1886.3–2185.9)		13.5	(13.1–13.8)		24.6	(24.0–25.5)		7.8	(7.5–7.9)		61.8	(60.7–62.7)	
Frequency of eating breakfast																	
Twice a week or less	4	4.3%	2027.0	(1864.6–2121.1)	0.283	13.3	(12.8–13.8)	0.937	23.9	(23.7–25.2)	0.947	7.5	(7.4–7.9)	0.862	62.6	(61.1–63.3)	0.921
3-4 days a week	8	8.7%	2062.1	(1954.8–2161.5)		13.4	(13.1–13.5)		24.6	(24.1–25.3)		7.7	(7.6–7.9)		62.1	(61.2–62.6)	
5-6 days a week	10	10.9%	1966.9	(1892.1–2192.8)		13.6	(12.5–13.8)		24.9	(23.7–25.6)		7.7	(7.5–7.9)		61.8	(60.7–62.5)	
Daily	70	76.1%	2105.3	(1981.1–2300.2)		13.3	(12.9–13.6)		24.5	(23.8–25.8)		7.7	(7.4–8.1)		62.2	(60.6–63.1)	
BMI																	
18.5~25.0	53	57.6%	2159.3	(1981.1–2300.2)	0.245	13.2	(12.8–13.6)	0.311	24.5	(23.7–25.8)	0.951	7.7	(7.4–8.0)	0.631	62.2	(60.8–63.3)	0.743
25.0~30.0	37	40.2%	2029.7	(1942.7–2222.3)		13.4	(13.1–13.8)		24.7	(23.9–25.6)		7.7	(7.5–8.1)		61.9	(60.8–62.9)	
30.0~35.0	2	2.2%	2001.7	(1892.1–2111.2)		13.5	(12.6–14.3)		24.8	(23.6–25.9)		7.6	(7.3–7.9)		61.8	(59.8–63.7)	
Grade																	
Freshman	34	37.0%	2205.2	(1992.5–2478.6)	0.031^†^	13.2	(12.5–13.5)	0.257	25.0	(24.0–26.7)	0.104	7.8	(7.5–8.3)	0.262	61.7	(60.3–62.8)	0.236
Sophomore	32	34.8%	2018.9	(1932.1–2231.8)		13.3	(12.8–13.7)		24.3	(23.5–25.4)		7.6	(7.4–8.0)		62.4	(61.0–63.6)	
Junior	26	28.3%	2022.5	(1947.3–2216.0)		13.5	(13.1–13.7)		24.9	(23.8–25.8)		7.8	(7.5–8.1)		62.1	(60.6–62.9)	

*p-value: Kruskal-Wallis test; multiple comparisons of pairs were performed using the Bonferroni method. †A significant difference was observed among the three groups, but the difference was weak in multiple comparisons (freshmen > sophomores, *p* < 0.057). a and b: The results of multiple comparisons showed a significant difference between the second and fourth teams (^a^*p*<0.01, ^b^*p* < 0.0).

**Table 3.** Association between performance level and nutrient intake.

	All (n = 92)		First team (n = 12)		Second team (n = 14)		Third team (n = 34)		Fourth team (n = 34)		
	Median	(Interquartile Range)	Median	(Interquartile Range)	Median	(Interquartile Range)	Median	(Interquartile Range)	Median	(Interquartile Range)	*p*
Water, g	2433.4	(2295.5–2582.3)	2477.2	(2372.4–2535.0)	2589.5	(2364.8–2885.9)	2456.5	(2333.1–2545.1)	2350.3	(2234.9–2530.1)	0.026
Protein, g	79.0	(76.1–84.2)	80.3	(73.9–82.7)	82.6	(77.9–87.3)	78.5	(76.6–86.3)	78.3	(74.9–79.8)	0.128
Sum of amino acid residues	68.6	(66.2–72.9)	69.7	(64.3–71.9)	71.8	(67.7–75.6)	68.3	(66.6–74.8)	68.2	(65.2–69.3)	0.134
Total fat, g	60.9	(57.7–66.2)	60.5	(56.4–63.4)	63.2	(59.3–68.7)	62.3	(58.8–68.8)	59.2	(56.6–62.8)	0.053
Saturated fatty acid, g	17.6	(16.5–19.1)	17.1	(16.2–18.3)	18.4	(17.2–20.0)	18.1	(16.8–19.5)	17.1	(16.1–18.0)	0.021^a^
Monounsaturated fatty acid, g	22.9	(21.4–25.2)	22.6	(21.0–23.7)	23.6	(22.5–25.3)	23.8	(22.1–26.4)	21.8	(21.0–23.8)	0.047^a^
Polyunsaturated fatty acid, g	13.2	(12.7–14.0)	13.0	(12.6–13.8)	13.4	(13.0–14.2)	13.4	(12.9–14.7)	13.1	(12.7–13.5)	0.159
n-3 PUFA	2.8	(2.7–2.9)	2.8	(2.7–2.9)	2.8	(2.7–2.9)	2.8	(2.7–3.0)	2.8	(2.7–2.9)	0.501
n-6 PUFA	10.4	(9.9–11.0)	10.2	(9.8–10.9)	10.6	(10.2–11.3)	10.5	(10.1–11.7)	10.3	(9.9–10.5)	0.105
Triacylglycerol equivalents, g	56.2	(53.1–61.1)	55.7	(51.9–58.4)	58.3	(54.7–63.5)	57.5	(54.2–63.7)	54.6	(52.1–57.8)	0.051
Cholesterol, mg	329.8	(319.3–349.4)	336.7	(317.5–347.9)	336.7	(320.2–348.5)	327.7	(320.5–353.1)	328.8	(318.6–339.5)	0.743
Carbohydrate, g	289.8	(265.4–323.8)	287.4	(265.2–326.2)	331.6	(313.3–372.7)	281.8	(270.7–316.5)	285.4	(251.9–308.5)	0.004^b^
Total dietary fiber, g	12.8	(11.8–14.3)	12.2	(11.7–13.5)	14.4	(12.6–17.5)	12.8	(12.0–14.1)	12.6	(11.4–14.1)	0.069^c^
Water soluble fiber, g	2.8	(2.6–3.1)	2.7	(2.6–2.8)	3.1	(2.8–4.2)	2.8	(2.6–3.1)	2.7	(2.5–3.0)	0.140
Water insoluble fiber, g	9.4	(8.6–10.5)	9.0	(8.5–10.2)	10.7	(9.3–12.0)	9.3	(8.9–10.4)	9.2	(8.3–10.5)	0.045^d^
Sodium, mg	4175.8	(4001.8–4436.2)	4206.0	(4000.0–4419.1)	4349.2	(4103.1–4702.9)	4188.3	(4022.1–4524.5)	4119.6	(3937.5–4257.2)	0.084
Potassium, mg	2626.2	(2437.2–2883.4)	2646.4	(2412.9–2805.4)	2836.4	(2510.2–3165.3)	2631.5	(2466.0–2925.9)	2536.2	(2422.3–2758.3)	0.100
Calcium, mg	523.8	(504.1–550.3)	512.8	(506.3–544.9)	544.5	(516.5–615.5)	532.7	(512.8–552.9)	511.8	(501.0–538.3)	0.017^d^
Magnesium, mg	280.2	(260.9–308.0)	279.5	(259.2–299.5)	313.4	(278.1–339.6)	278.4	(260.6–319.3)	279.0	(252.9–298.9)	0.086
Phosphorus, mg	1125.5	(1072.0–1223.6)	1127.8	(1041.6–1202.4)	1211	(1106.8–1296.7)	1117.2	(1080.2–1274.4)	1122.1	(1055.4–1169.8)	0.083
Iron, mg	8.6	(8.1–9.3)	8.6	(8.1–9.1)	9.1	(8.5–9.8)	8.4	(8.1–9.5)	8.6	(7.9–9.1)	0.194
Zinc, mg	9.3	(8.7–10.3)	9.4	(8.3–9.9)	10.3	(9.5–10.9)	9.2	(8.7–10.5)	9.1	(8.3–9.7)	0.021^d^
Copper, mg	1.3	(1.2–1.4)	1.3	(1.2–1.4)	1.5	(1.3–1.5)	1.3	(1.2–1.4)	1.3	(1.1–1.4)	0.019^d^
Manganese, mg	4.3	(3.9–4.8)	4.0	(3.8–4.6)	4.9	(4.4–5.3)	4.2	(4.0–4.6)	4.3	(3.7–4.8)	0.043^d^
Iodine, µg	2056.9	(1944.8–2102.0)	2016.3	(1934.7–2100.5)	1949.2	(1649.5–2074.5)	2057.5	(1941.1–2091.5)	2092.9	(2008.8–2107.6)	0.047
Selenium, µg	87.6	(85.2–90.5)	88.4	(84.3–90.5)	89.0	(85.0–93.4)	87.9	(85.5–92.3)	86.6	(85.1–89.0)	0.411
Chromium, µg	9.2	(8.8–10.0)	9.4	(8.8–9.8)	9.5	(8.8–11.2)	9.4	(8.8–10.4)	8.9	(8.6–9.6)	0.172
Molybdenum, µg	221.6	(191.2–254.8)	223.0	(172.4–264.9)	254.3	(227.1–310.5)	220.0	(193.6–250.6)	214.2	(179.9–234.8)	0.018^d^
Retinol, µg	294.7	(276.4–334.6)	309.1	(286.9–325.6)	305.2	(274.2–343.6)	292.9	(276.6–338.5)	291.9	(276.3–317.3)	0.819
Alpha-carotene, µg	509.8	(477.7–537.6)	484.2	(475.6–522.7)	517.1	(483.3–603.6)	514.7	(482.3–528.8)	488.6	(474.9–537.6)	0.535
Beta-carotene, µg	2783.2	(2617.8–2929.0)	2690.6	(2616.8–2857.5)	2924.4	(2658.2–3244.3)	2801.2	(2665.9–2878.2)	2753.3	(2604.9–2894.4)	0.470
Beta-cryptoxanthin, µg	215.1	(112.6–381.7)	242.5	(196.4–400.7)	279.1	(111.9–656.2)	214.3	(112.1–374.4)	213.7	(112.6–253.1)	0.356
Beta carotene equivalents, µg	3310.3	(3102.8–3521.0)	3269.4	(3100.1–3515.2)	3580.4	(3124.7–3926.9)	3348.9	(3162.0–3480.6)	3295.2	(3076.9–3474.9)	0.506
Retinol equivalents, µg	620.0	(567.6–689.1)	642.3	(596.3–668.6)	649.4	(583.8–710.4)	599.5	(568.1–693.1)	609.6	(567.3–654.9)	0.678
Vitamin D, µg	9.7	(9.1–10.2)	9.7	(9.1–10.4)	9.6	(9.4–10.1)	9.4	(9.1–10.4)	9.7	(9.2–10.0)	0.989
Alpha-tocopherol, mg	7.9	(7.5–8.6)	7.8	(7.4–8.3)	8.1	(7.8–9.1)	8.0	(7.6–8.6)	7.7	(7.4–8.4)	0.234
Beta-tocopherol, mg	0.4	(0.3–0.4)	0.4	(0.3–0.4)	0.4	(0.3–0.4)	0.4	(0.3–0.4)	0.4	(0.3–0.4)	0.152
Gamma-tocopherol, mg	11.0	(10.7–11.5)	10.8	(10.6–11.4)	11.0	(10.9–11.8)	11.1	(10.8–11.9)	10.9	(10.5–11.1)	0.118
Delta-tocopherol, mg	2.9	(2.8–3.1)	2.8	(2.8–3.1)	2.9	(2.8–3.3)	2.9	(2.8–3.2)	2.8	(2.8–3.0)	0.297
Vitamin K, µg	222.0	(203.3–255.1)	206.9	(193.8–224.1)	245.1	(203.5–288.6)	225	(207.1–283.7)	220.5	(200.2–244.4)	0.241
Vitamin B_1_, mg	1.2	(1.1–1.4)	1.2	(1.0–1.3)	1.3	(1.2–1.6)	1.2	(1.1–1.3)	1.2	(1.0–1.3)	0.100
Vitamin B_2_, mg	1.5	(1.4–1.7)	1.5	(1.4–1.6)	1.6	(1.4–1.8)	1.5	(1.4–1.8)	1.5	(1.4–1.5)	0.119
Niacin, mg	20.1	(18.9–21.7)	19.8	(18.7–21.1)	20.3	(20–24.1)	19.8	(18.9–21.9)	20.1	(19.0–21.5)	0.466
Vitamin B_6_, mg	1.5	(1.3–1.8)	1.5	(1.2–1.6)	1.7	(1.5–2.1)	1.4	(1.3–1.8)	1.5	(1.3–1.7)	0.170
Vitamin B_12_, µg	8.3	(7.7–8.9)	8.2	(7.7–8.9)	8.5	(7.7–9.0)	8.2	(7.7–8.9)	8.2	(7.8–8.7)	0.905
Folate, µg	359.8	(335.2–401.6)	360.3	(333.2–384.1)	396.1	(351.6–443.6)	357.5	(336.3–408.7)	351.7	(331.4–388.8)	0.277
Pantothenic acid, mg	6.3	(5.8–6.9)	6.3	(5.7–6.8)	7.1	(6.1–7.6)	6.2	(5.9–7.3)	6.2	(5.7–6.5)	0.070
Biotin, µg	45.3	(43.9–48.8)	45.8	(44.1–47.9)	47.7	(44.9–49.9)	44.9	(43.9–49.6)	45.4	(43.7–46.5)	0.381
Vitamin C, mg	102.3	(93.2–126.7)	104.5	(97.0–116.4)	112.9	(94.3–164.2)	105.5	(91.4–129.4)	97.7	(93.0–118.6)	0.512
Daidzein, mg	10.5	(9.5–12.1)	10.7	(10.3–11.0)	11.4	(9.8–13.0)	10.4	(9.6–12.6)	10.3	(9.1–11.8)	0.264
Genistein, mg	17.7	(16.1–20.4)	18.1	(17.6–18.4)	19.6	(16.7–21.8)	17.7	(16.4–21.3)	17.3	(15.5–19.7)	0.235
Lycopene, µg	1880.9	(1877.0–1989.9)	1879.9	(1879.2–2013.2)	1939.4	(1878.7–2125.7)	1883.4	(1877.3–1989.2)	1879.3	(1875.5–1889.6)	0.276
Ethanol, g	5.6	(5.6–5.6)	5.6	(5.6–8.0)	5.6	(5.6–5.6)	5.6	(5.6–5.6)	5.6	(5.6–5.6)	0.551

*p*-value: Kruskal-Wallis test; multiple comparisons of pairs were performed using the Bonferroni method.

^a^As a result of multiple comparisons, the figures for third team were higher than those for fourth team 4, but the difference was minimal (*p* < 0.10).

^b^The results of the multiple comparisons were second team > third team (*p* < 0.05) and second team > fourth team (*p* < 0.01).

^c^and ^d^Similarly, second team > fourth team (c*p* <0.05, d*p* < 0.10).

**Table 4.** Association between performance level and food intake.

	All (n = 92)		First team (n = 12)		Second team (n = 14)		Third team (n = 34)		Fourth team (n = 34)		
	Median	(IQR)	Median	(IQR)	Median	(IQR)	Median	(IQR)	Median	(IQR)	*p*
Cereals, g	515.2	(457.8–568.1)	495.8	(452.1–596.4)	581.8	(545.3–610.0)	510.2	(457.5–553.4)	508.2	(451.3–541.9)	0.011 ^a^
Potatoes and starches, g	37.8	(35.5–39.8)	38.7	(36.6–39.4)	38.2	(35.5–40.4)	38	(35.8–40.6)	36.1	(35.5–39.0)	0.252
Sugar, g	5.5	(5.5–5.6)	5.5	(5.5–5.5)	5.6	(5.5–5.8)	5.5	(5.5–5.6)	5.5	(5.5–5.5)	0.007 ^b^
Pulses, g	55.8	(52.6–64.4)	57.2	(53.9–64.3)	63.3	(53.9–71.3)	55.3	(53.4–64.1)	54.4	(52.0–58.9)	0.116
Nuts and seeds, g	2.5	(2.5–2.5)	2.5	(2.5–2.5)	2.5	(2.5–2.6)	2.5	(2.5–2.5)	2.5	(2.5–2.5)	0.452
Vegetables, g	266.3	(253.9–291.5)	260.8	(250.2–277.8)	285.8	(261.4–318.0)	269.4	(259.5–298.5)	257.1	(247.3–275.7)	0.050
Green and yellow, g	100.5	(98.6–114)	98.4	(100.3–108.4)	107.7	(100.1–122.3)	101.1	(99.3–112.0)	100.3	(97.9–105.2)	0.411
White vegetables, g	165.4	(159.2–180.5)	159.4	(157.3–171.1)	174.3	(161.1–182.4)	172.1	(163.6–184.8)	162.5	(156.8–175.9)	0.034
Pickles, g	12.8	(12.7–12.9)	12.8	(12.7–12.9)	12.8	(12.7–12.9)	12.8	(12.7–12.9)	12.7	(12.7–12.8)	0.469
Fruits, g	86.2	(35.7–128.2)	101.0	(61.5–131.7)	109.2	(43.2–313.2)	94.6	(33.7–126.9)	51.5	(31.7–116.7)	0.220
Fungi, g	15.5	(15.5–17.1)	15.9	(15.5–17.1)	15.5	(15.5–16.9)	16.0	(15.5–17.0)	15.5	(15.5–16.9)	0.675
Algae, g	6.2	(5.2–8.3)	6.5	(5.8–7.5)	7.4	(5.3–12.8)	6.6	(5.2–10.3)	5.6	(5.2–7.0)	0.133
Fish and shellfish, g	90.2	(87.2–94.7)	91.2	(88.5–94.3)	89.2	(88.3–94.7)	90.1	(87.3–94.6)	90.2	(86.7–94.7)	0.934
Meat, g	91.5	(83.7–105.1)	88.2	(80.0–102.1)	96.5	(88.2–106.7)	96.8	(87.5–105.9)	87.5	(82.6–101.2)	0.355
Eggs, g	37.9	(36.5–40.3)	37.9	(36.7–41.1)	37.9	(36.7–38.8)	37.9	(36.5–40.9)	37.9	(36.7–40.3)	0.904
Milk and dairy products, g	94.2	(88.9–109.0)	90.9	(89.7–107.4)	107.2	(93.6–160.7)	98.1	(90.7–114.1)	92.0	(86.9–101.1)	0.025 ^a^
Fats and oils, g	10.7	(10.1–11.9)	10.4	(9.9–11.6)	11.2	(10.4–12.5)	11.0	(10.4–12.1)	10.4	(10.0–11.3)	0.180
Confectionaries, g	24.2	(21.7–28.3)	24.1	(23.2–27)	23.5	(21.8–30.5)	25.3	(23.1–28.2)	22.8	(21.7–25.9)	0.257
Alcoholic beverages, g	91.0	(91.0–91.0)	91.0	(91.0–110.2)	91.0	(91.0–91.0)	91.0	(91.0–91.0)	91.0	(91.0–91.0)	0.573
Non-alcoholic beverages, g	491.1	(453.3–550.9)	474	(442.8–546.9)	508.2	(473.6–584.0)	508.3	(476.8–556.2)	484.7	(435.1–524.1)	0.188
Seasonings and spices, g	124.5	(113.8–138.6)	129.7	(117.3–143.3)	121.4	(117.1–161.0)	129.7	(117.3–144.4)	118.7	(110.1–131.6)	0.121

*p*-value: Kruskal-Wallis test; multiple comparisons of pairs were performed by the Bonferroni method.

a and b: The results of multiple comparisons showed a significant difference between the second and fourth teams (^a^*p* < 0.05, ^b^*p* < 0.01).

## 3. Results

### 3.1. Study population

The participants’ characteristics are shown in Table 1. Of the 92 participants, 34 (37.0%) were freshmen, 32 (34.8%) were sophomores, and 26 (28.3%) were juniors. Regarding participants’ performance levels, 12 (13.0%) played in the first team, 14 (15.2%) in the second team, 34 (37.0%) in the third team, and 32 (34.8%) in the fourth team.

### 3.2. Energy intake and the balance of energy production ratios

Table 2 shows the associations of the participants’ characteristics, performance levels, and other variables with energy intake and the balance of energy production ratios. The living arrangement, frequency of eating breakfast, or body mass index (BMI) bore no association with energy intake or the balance of energy production ratios. Energy intake differed among different performance levels and grades: it was higher in the second team (*Mdn***=**2304.2 kcal) compared with the fourth team (*Mdn***=**2009.3 kcal) and it was slightly higher in the freshmen (*Mdn***=**2205.2 kcal) than in the sophomores (*Mdn***=**2018.9 kcal). The *p*-value between the second team and fourth team was *p* < 0.01. A significant difference existed among the three grade groups, but the difference was weak in multiple comparisons (freshmen > sophomores, *p* < 0.057). The median energy intake of the first team was 2055.1 kcal, which was not significantly different from that of the other teams. Protein ratios differed among different performance levels and were significantly higher in the fourth team compared with the second team.

### 3.3. Performance levels and nutrient intake

The association of performance levels with nutrient intake is shown in Table 3. Significant differences were observed in the intake of carbohydrate (*p* = 0.004), calcium (*p* = 0.017), zinc (*p* = 0.021), copper (*p* = 0.019), manganese (*p* = 0.043), saturated fatty acid (*p* = 0.021), monounsaturated fatty acid (*p* = 0.047), insoluble dietary fiber (*p* = 0.045), iodine (*p* = 0.047), and molybdenum (*p* = 0.018) among the different performance levels. In the multiple comparison, carbohydrate intake was higher in the second team compared with the third (*p* = 0.031) and fourth teams (*p* = 0.002). The intake of saturated fatty acid (*p* = 0.066) and monounsaturated fatty acid (*p* = 0.134) was higher in the third team compared with the fourth team but the difference was weak. The intake of water-insoluble fiber (*p* = 0.047), calcium (*p* = 0.037), zinc (*p* = 0.016), copper (*p* = 0.011), iodine (*p* = 0.042), and molybdenum (*p* = 0.010) was higher in the second team than in the fourth team. Manganese also showed a similar trend, but the difference was not significant (*p* = 0.064). In terms of these nutrient intakes, the intake of the first team did not differ significantly from that of the other teams.

### 3.4. Performance levels and intake of each food group

The associations of performance levels and the intake of each food group are shown in Table 4. The intake of grain, sugar, and dairy differed among the different performance levels and was significantly greater in the second team than in the fourth team. The first team did not show a significant difference, compare with the other teams.

## 4. Discussion

This cross-sectional study investigated the associations of nutritional intake status with performance levels and characteristics/living conditions by surveying the diet and habits of college-level baseball players using the FFQ. The study population had a large deficiency in energy. Comparison of its nutritional intake with the recommended dietary allowances (RDAs) of the Dietary Reference Intakes for Japanese, 2020 [21] revealed deficiencies in protein, fat, and carbohydrate associated with energy deficiency. The RDAs is the daily intake that is estimated to meet the daily needs of most (97%-98%) people belonging to a particular group. Moreover, dietary fiber and calcium were also deficient. The intake of food groups such as potatoes, beans, vegetables, fruits, eggs, dairy products, and fats was small. The second team had significantly more intakes of carbohydrate, calcium, and insoluble dietary fiber.

Energy intake was significantly higher in the second team compared with the fourth team, with an overall median of 2,077 kcal/day. According to the Dietary Reference Intakes for Japanese, 2020 [21], the estimated energy requirement for men aged 18–29 years with an activity level of III (high) is 3,050 kcal/day and the estimated energy requirement per body weight is 3,555 kcal (the participants’ median weight was 75 kg). A previous study on NCAA Division I baseball players found a daily energy requirement of 3,558 kcal for baseball players [22]. Of the 92 participants in the present study, only 4 consumed 3,000 kcal or more, indicating that the energy intake of the study population was considerably small. There are many reports of energy deficiency in athletes [22–24], similar to the results of the present study. A nutritionally balanced diet is important, and the first step in achieving it is to ensure that the energy intake is commensurate with the energy expenditure. Chronic energy deficiency leads to depletion in muscle mass, muscle strength, and bone density. However, the present study results need to be investigated in further detail because participants’ self-reported dietary intake may differ from their actual intake.

The requisite balance of energy production ratios is 13–20% for protein, 20–30% for fat, and 50–65% for carbohydrate [25], and the study population generally met this balance. The protein ratio was significantly higher at 13.5% in the fourth team compared with 12.9% in the second team. Overall, it was 13% in the entire cohort, which corresponds to the lower limit. According to the International Society of Sports Nutrition, the reference protein intake is 1.4–2.0 g/kg body weight/day [25], which corresponds to 105–150 g in the study population (using a median body weight of 75 kg, as above) (it was also 1.5–2.0/kg body weight/day in other studies). The median protein intake for the entire study population was found to be 79.0 g, which was considerably small. Protein is essential for the enhancement of muscles in athletes. Muscle proteins are broken down during exercise and synthesized after exercise. All essential amino acids, as well as all proteinogenic amino acids, are necessary for muscle protein synthesis. Fat is a nutrient that enables efficient energy metabolism. One gram of fat contains 9 kcal of energy. The intake of saturated fatty acid and monounsaturated fatty acid was significantly higher in the third team compared with the fourth team. Body fat that negatively affects athletic performance needs to be reduced, and excessive intake of fat needs to be avoided because fat accounts for a small proportion of the energy source in baseball, a sport which mainly relies on the use of instantaneous force. Based on the present study’s finding, the ratio of saturated fatty acid to energy intake tended to be high. A recent study reported an association between *n*-3 fatty acid intake and improved athletic performance [26]. However, *n*-3 fatty acid are integral nutrients for cellular health and development, neuronal activity, cognitive function, and even muscle development. In current study’s population was deficient in protein, fat, and carbohydrate as well as energy. Meat is an important source of energy and protein, although some cuts are higher in saturated fatty acids than others. Actively consuming protein sources, including meat, is important.

Carbohydrate intake was significantly higher in the second team compared with the third and fourth teams. Carbohydrate (sugar) play a crucial role in exercise. The recommended carbohydrate intake for athletes ranges from 6–10 g/kg body weight/day [27], which corresponds to 450–750 g for the study population, whose median body weight was 75 kg. However, the overall median carbohydrate intake in this population was 289.8 g, which is considerably lower than the recommended amount. The carbohydrate intake in the second team, which was significantly greater than that in the third and fourth teams, was 331.6 g. Carbohydrate deficiency decreases the amount of stored glycogen and limits the amount of energy available during exercise, resulting in decreased athletic performance. Carbohydrates constitute the main energy source, particularly in sports that require a continuous supply of energy; therefore, it is important to eat meals concentrated in carbohydrates habitually to increase the amount of glycogen in the muscles and liver. Moreover, supplementation with sugar immediately after exercise helps not only to store glycogen in the muscles but also to inhibit muscle protein degradation.

Insoluble dietary fiber intake was significantly higher in the second team compared with the fourth team. The overall dietary fiber intake was 12.8 g, well short of the target amount of 21 g established in the Dietary Reference Intakes (an amount that aimed to prevent the development of lifestyle diseases). The target amount in the United States is even larger at 14 g/1,000 kcal [28]. A recent study also reported that short-chain fatty acid generated by the gut microbiota feeding on dietary fiber was associated with increased muscle function and glycogen in skeletal muscles [29]. However, attention should be paid to timing, because ingestion before or after exercise may lead to gastrointestinal problems.

Calcium intake was significantly higher in the second team at 545 mg compared with 512 mg in the fourth team. The overall median calcium intake was 523.8 mg, which is less than the RDA of 800 mg and even below the estimated average requirement (EAR) of 650 mg/day, implying that the requirements of half of the study population were not met. For athletes, 1,000 mg/day of calcium is recommended, taking into account the increased metabolism and bone mass due to exercise [1]. Calcium performs important physiological functions by regulating muscle contraction and participating in myocardial function. Bone stimulation through exercise increases bone density. However, excessive training and continued inadequate calcium intake from meals decrease bone density. Athletes should be aware of calcium-rich foods and try to incorporate them in their daily diet.

In this study, vitamin B1 intake was at the lower limit of the EAR and half of the population was deficient. Athletes were likely deficient in the entire vitamin B-complex. The vitamin B-complex performs functions such as energy production, absorption and transport of iron, and blood cell production. Therefore, its deficiency should be avoided.

When the intake of each food group was compared with the food composition for men aged 18–29 years with high physical activity levels [30], deficiencies in potatoes, beans, vegetables, fruits, eggs, milk, and fats were found. Potatoes contain high levels of carbohydrates, dietary fiber, vitamin C, and minerals. Their large energy content makes them an effective supplemental dietary component. Many beans contain a good balance of the vitamin B complex, minerals, and dietary fiber. Soybeans contain a large amount of vitamin B1, which is important for carbohydrate metabolism. Moreover, they are rich in protein, especially lysine, an essential amino acid. Because rice(a staple food) contains a small amount of lysine, eating it with soybeans provides an effective amino acid score. The recommended intake of vegetables is 350 g/day. However, their intake was small at 266.3 g. It is beneficial to ingest green and yellow vegetables and light-colored vegetables in a 1:2 ratio. However, the intake of light-colored vegetables was 165.4 g. Light-colored vegetables contain copious amounts of dietary fiber, and many of them such as lettuce, cabbage, cucumbers, and daikon radishes can easily be eaten raw and should be consumed actively.

Although the recommended intake of fruits is 200 g/day, our participants consumed only 86.2 g/day. Fruits are refreshing despite their large energy content. Therefore, we think that it is effective to consume them after meals or between meals. Vitamin C, which is present in fruits and vegetables in great abundance, has excellent antioxidant capacity and can reduce oxidative stress caused by exercise. The recommended amount of egg consumption is 55 g/day (approximately 1 egg). Eggs contain a large amount of protein and have a high amino acid score. They are also better sources of fatty acids and other bioactive compounds (e.g. choline, betaine, lutein, zeaxanthin) that could offer athletic and health-promoting benefits when consumed in adequate amounts. They are easy to cook and are good source of protein and other nutrients. The dairy intake was significantly higher at 107 g in the second team compared with 92 g in the fourth team. The overall median was 94 g, well below the reference level of 380 g. Dairy products such as milk, yogurt, and cheese contain a large amount of calcium, and characteristically, calcium is absorbed better from dairy products compared with plant-based foods. Dairy products also contain a large amount of other nutrients such as protein, fat, and vitamin B1. Adding milk to each meal is considerably expected to improve nutrient deficiency. Milk consumption acutely increases muscle protein synthesis, thereby improving the net muscle protein balance. Furthermore, when postexercise milk consumption is combined with resistance training, greater increases in muscle hypertrophy and lean mass have been observed [31]. Athletes who live alone and do not usually cook may find it difficult to prepare meals containing sufficient energy. We suggest that such athletes inculcate the habit of drinking milk.

The overall median fat intake was 10.7 g, well below the recommended amount of 30 g. Although excessive fat consumption should be avoided, it can effectively increase total energy; thus, fat intake needs to be increased in college-level athletes, so long as the balance of energy production ratio is maintained.

The present study surveyed the eating habits of college baseball players using the FFQ and identified several issues. The intake of many items by the second team were significantly greater than that of the lower performance groups. However, all groups had inadequate intake of many nutrients and foods. They were energy deficient with associated deficiencies in protein, fat, carbohydrates, dietary fiber and calcium. The intake of food groups, such as potatoes, beans, vegetables, fruits, eggs, dairy products, and fat was insufficient. To avoid energy and nutrient deficiencies, we recommend that each meal include a staple food, main dish, side dishes, milk, and fruit. Moreover, the timing of carbohydrate intake is also important. Before exercise, for a stable and sustained energy supply, a recommendation is to consume complex carbohydrates that are high in starch and dietary fiber and consume low glycemic index (GI) foods that slowly raise blood sugar levels. Consuming foods high in simple carbohydrates such as glucose and fructose, which are effective in maintaining performance, and high GI foods is desirable [1]. The same is true for protein: consuming an appropriate amount of protein before exercise can help prevent muscle breakdown during exercise and may also provide a source of energy when needed. It is believed that protein intake after exercise is also likely to contribute to muscle repair and increase muscle mass [1]. Previous research has suggested that a well-balanced diet comprising a staple food, main dish, and side dishes for three meals a day may prevent heat stroke [32]. Improving the nutritional status of athletes is also an important issue for the safe conduct of competitions. In order to improve competitive ability, it is important for athletes to first understand nutritional intake from their current eating habits. Further improvement can be expected by raising their awareness of this issue and providing institutional support.

The baseball team surveyed in the present study belongs to the first division of a prefectural college baseball league. Approximately 20% of the team members, who hail from all over the country, continue to play baseball after graduation, and the team contributes one player to the Nippon Professional Baseball Organization approximately every five years. Thus, the team is highly competitive. In general, the present study found that the second team’s players tended to have higher intake of energy, protein, and other nutrients. We posit that this is probably because they aspired and were motivated to make the first team. Overall, the fourth team’s players were deficient in multiple items. This may have resulted from lower activity levels due to the inclusion of injured players. The first team was not significantly different from the other teams in energy intake, nutrient intake, or food group intake, suggesting that they were not actively taking nutrients. This finding is likely because of the fact that the first team was playing regularly and was more focused on maintaining rather than improving their condition. This factor is especially true because this survey was conducted shortly after the end of the autumn college baseball league season. However, because these players are essentially participating in the game, it may be advisable that they increase their overall nutrition intake.

To the best of our knowledge, these findings are novel, and further research is desired in the future. Moreover, the fact that many dietary insufficiencies were observed even among college baseball players from such a club is a concern because it suggests low awareness of diet and inadequate education and support. An overall lack of intake was noted, and organized and strategic remedial measures need to be implemented to improve this problem, rather than relying on individual efforts.

The present study surveyed the nutritional intake status of active college baseball players using the FFQ and evaluated the characteristics of their nutritional intake status in terms of performance and other aspects. When interpreting the data, several limitations must be considered. First, the participants in the present study were recruited from a single sports club, limiting the sample size. It is difficult to generalize the results to all baseball players, and further research is recommended. Second, the data obtained were based on participants’ own report. This may have introduced bias and affected the quality of the data. Third, we did not investigate the differences between positions. The amount of exercise in each baseball position is expected to be different. We would like to make this factor an issue in future research. Fourth, the present study did not enquire about the intake of supplements, including supplementary foods, and thus, its impact could not be evaluated. Future studies should examine the nutritional intake status of the athletes from all perspectives. Finally, generalizability is limited because this study involved only one university.

For future research, we are also considering using blood tests to investigate the nutritional status of student athletes in detail. We believe that better performance would be achieved if we can determine the nutritional components required by individuals and approach nutrition improvement by using that information.

## 5. Conclusions

This study demonstrated the presence of deficiencies in the amount of energy and nutrients, such as protein, fat, and carbohydrate in college baseball players. It also suggested inadequate intake of potatoes, beans, vegetables, fruits, eggs, dairy products, and fat as well as dietary fiber and calcium. In order to overcome these deficiencies, organized and strategic remedial measures are needed, instead of relying upon individual-level efforts alone. Differences in the intake of carbohydrate, calcium, and insoluble dietary fiber among different performance levels were observed, with significantly higher intake of carbohydrate, calcium, and insoluble dietary fiber in the second team. Therefore, further studies examining these factors are warranted. Nonetheless, this study provides useful information for the development of strategies to support nutritional intake in college baseball players.
